# A cross-sectional epidemiological study of the relationship between sleep duration, quality, and rhythm and presenteeism in workers

**DOI:** 10.1007/s41105-021-00339-4

**Published:** 2021-07-08

**Authors:** Osamu Itani, Yoshitaka Kaneita, Yuichiro Otsuka, Mikiko Tokiya, Maki Jike, Yuuki Matsumoto, Sachi Nakagome, Yu Kinoshita

**Affiliations:** 1 Division of Public Health, Department of Social Medicine, Nihon University School of Medicine, Itabashi-ku, Tokyo Japan; 2 Department of Public Health and Epidemiology, Faculty of Medicine, Oita University, Yufu-city, Oita Japan; 3 Department of Food Safety and Management, Faculty of Food and Health Sciences, Showa Women’s University, Setagaya-ku, Tokyo Japan

**Keywords:** Sleep duration, Insomnia, Occupational health, Health management, Observational study

## Abstract

This study aims to examine the relationship of sleep (sleep duration, sleep quality, and sleep rhythm) with presenteeism in workers while controlling for other confounding factors. A total of 2375 workers of six Japanese companies received self-administered questionnaires from June to November 2018. Information on sleep duration was used to evaluate sleep quantity, the Athens Insomnia Scale (AIS) was used to evaluate sleep quality, and workers’ engagement in shift work was used to determine their sleep rhythms. We used the World Health Organization Health and Work Performance Questionnaire to evaluate presenteeism. Information on lifestyle (exercise, smoking, etc.), sex, and age was also collected. We conducted a logistic regression analysis with high absolute/relative presenteeism as an objective variable, sleep duration, AIS, and shift work as dependent variables, and basic attributes and lifestyle factors as adjustment factors. Completed questionnaires were collected from 1992 workers (aged 18–79 years; 25.2% women; response rate: 83.9%). Logistic regression analysis showed that high absolute presenteeism was significantly associated with poor sleep quality (high AIS score; *P* < 0.001) but not with sleep duration (*P* = 0.326) and shift work (*P* = 0.177). High relative absenteeism was significantly associated with poor sleep quality (high AIS score; *P* = 0.001) but not with sleep duration (*P* = 0.461) or shift work (*P* = 0.245). We showed that poor sleep quality is significantly associated with a high level of presenteeism. This suggests focusing on improving sleep quality is important for reducing presenteeism among workers.

## Introduction

Japan’s Ministry of Economy, Trade, and Industry (METI) has been recognizing and publicizing companies that are strategically addressing workers’ health from a management perspective as “excellent health management corporations.” “Health and Productivity Management (H&PM)” is an approach that considers health management of employees from a corporate management perspective and promotes it strategically [1]. A company’s productivity can suffer as a result of workers being absent from work due to illness or other reasons. This is called absenteeism [2], and is a problem for corporate management. However, since it is a visible phenomenon, it is relatively easy to monitor and manage with countermeasures. By contrast, presenteeism occurs when workers arrive to work but are unable to perform adequately due to some physical or mental problem, resulting in a decline in productivity [3]. Presenteeism is difficult to recognize because workers are present at work and often appear well. Moreover, presenteeism is a major component of worker health costs incurred by companies [4] and is regarded as a difficult but important issue in promoting H&PM.

Before exploring ways of reducing presenteeism, it is first necessary to consider the various factors related to presenteeism. Previous epidemiological studies have established that presenteeism is significantly associated with medical conditions such as depression [5] and hypertension [6], as well as lifestyle factors such as exercise [7] and smoking [8]. Among the various components of workers’ lives, sleep is one that occupies a large proportion of a worker’s lifetime. The 2016 Survey on Time Use and Leisure Activities, for which participants were randomly sampled nationwide, reported that workers’ average daily sleep duration is 7 h 44 min for men and 7 h 25 min for women [9]. Sleep is, therefore, a major factor in workers’ lives.

Several previous epidemiological studies have linked sleep with presenteeism, but had significant limitations that cannot be ignored. Although sleep duration has been used as a quantitative measure of sleep in previous studies [10–12], sleep has non-quantitative aspects as well. One epidemiological study on the relationship between sleep and health outcomes reported that when both quantitative and qualitative aspects of sleep were taken into account simultaneously, only sleep quality was reported to be significantly associated with health outcomes [13]. Furthermore, the disturbance of the circadian rhythm that is observed in shift workers is associated with the onset of health problems [14]. Thus, when evaluating the relationship between sleep and health outcomes, it is necessary to evaluate not only the quantitative aspect of sleep but also factors such as sleep quality and the circadian rhythm.

Therefore, we planned a new epidemiological study on the relationship between sleep and presenteeism in workers. To overcome the aforementioned limitations, we conducted a comprehensive evaluation of three sleep components: quantity (duration), quality, and rhythm. For the assessment of sleep quality, we used the Athens Insomnia Scale (AIS) [15]—a questionnaire with thoroughly verified validity and reliability. Moreover, we adjusted for confounding factors (e.g., exercise [7], smoking [8]) known to be associated with presenteeism. Thus, we aimed to obtain more epidemiologically valid results in comparison with previous studies.

## Materials and methods

### Participants and survey procedure

This was a cross-sectional observational study that surveyed the workers of six companies operating in different industries and located in various regions of Japan. All 2375 workers who agreed to participate in this study worked for these six companies, which consisted of two pharmaceutical companies (one of which has three drug manufacturing plants), an information technology company, a medical equipment wholesaler, a medical facility in the Kanto region, and an auto parts plant in the Kyushu region. Workers from each company were surveyed between June and November 2018. The questionnaires were distributed to the workers through the representatives of each company in succession. The workers were asked to fill out the questionnaires on their own. Completed questionnaires were collected through the representatives of the companies at a later date.

### Questionnaire

Self-administered questionnaires were distributed to the workers through company representatives and were subsequently completed by the workers. The questionnaire contained items pertaining to sleep duration, sleep quality, shift work, presenteeism, and various factors including professional duties and lifestyle.

To evaluate sleep duration (“What has been your average daily sleep duration over the past month?”), the participants were asked to choose one of the following options: < 5 h; ≥ 5 but < 6 h; ≥ 6 but < 7 h; ≥ 7 but < 8 h; ≥ 8 but < 9 h; ≥ 9 h. The Japanese version of the Athens Insomnia Scale [15, 16] was used as part of the survey questionnaire for the assessment of sleep quality. The AIS is a questionnaire designed to assess the severity of insomnia and is based on the International Classification of Disease, Tenth Revision [15]. It is composed of the following eight items: sleep induction, awakenings during the night, final awakening earlier than desired, total sleep duration (sufficient/slightly insufficient/markedly insufficient/very insufficient or did not sleep at all), overall quality of sleep, sense of well-being during the day, functioning (physical and mental) during the day, and sleepiness during the day. Each item is rated on a three-point scale, with the total score ranging from 0 to 24 points. According to a previous study, 4–5 points constitute a nocturnal cut-off value, whereas ≥ 6 points constitute a cut-off value for identifying pathological insomnia [16]. An item pertaining to shift work (“day work only” or “shift work”) was included to clarify whether the participants engaged in shift work.

Questions from the short-form Japanese edition [17] of the World Health Organization Health and Work Performance Questionnaire (WHO-HPQ) [18] were used to assess presenteeism. The WHO-HPQ is a self-administered questionnaire that was developed to assess lost productivity (presenteeism), sickness absenteeism, and crucial incidents (i.e., workplace successes and failures, occupational injuries, and workplace accidents) [19–22]. The HPQ Short Form (HPQ-SF) consists only of questions aimed at assessing presenteeism and absenteeism, has been translated into Japanese, and its reliability and validity have been verified [17]. Responses to three questions in the HPQ-SF are used to evaluate two types of presenteeism—absolute and relative. Absolute presenteeism is calculated as the difference between the score for oneself over the past 28 days and the score for the average worker in the same job. The absolute presenteeism score ranges from 0 (total lack of performance during time on the job) to 100 (no lack of performance during time on the job) [23]. That is, a lower absolute presenteeism score indicates poorer performance on the job and a higher level of absolute presenteeism. Relative presenteeism is the ratio of actual performance to the performance of most workers at the same job. The relative presenteeism score ranges from 0.25 to 2.0, where the lowest score (0.25) indicates the worst relative performance (25% or less of other workers' performance), and the highest score (2.0) indicates the best performance (200% or more of other workers' performance) [23]. That is, a lower relative presenteeism score indicates poorer performance relative to other workers and a higher level of relative presenteeism.

Other questionnaires items were used to collect information on the workers’ basic attributes (age, sex), type of work (desk/clerical), working position (non-managerial/managerial), type of employment (regular/irregular), smoking habit, drinking habit, exercise habit, overtime hours in 1 month, and holidays in 1 month. Moreover, the questionnaire included items from the Japanese Perceived Stress Scale (JPSS) [24, 25]. In addition, we used short form-8 (SF-8®) [26, 27], which is one of the most used health-related quality of life evaluation scales in the world, as an index to assess the medical condition (comorbidity) of the study population.

### Statistical analyses

The score distributions pertaining to absolute and relative presenteeism were tabulated. Absolute presenteeism scores were divided using a cut-off value of 40 points [28] and the association between absolute presenteeism scores and sleep duration was examined. The percentage distribution of each absolute presenteeism scores was tabulated and the association between absolute presenteeism scores (< 40, > 40) and sleep duration (< 5 h; ≥ 5 but < 6 h; ≥ 6 but < 7 h; ≥ 7 but < 8 h; ≥ 8 but < 9 h; ≥ 9 h) was assessed using the chi-squared test (including residual analysis). Missing data were excluded from the statistical analyses. Similarly, the cut-off point for relative presenteeism scores was set at 0.8 [28] and the association between relative presenteeism scores and sleep quality was examined. The relationship between absolute presenteeism scores (cut-off value, 40 points) and sleep quality was also examined. The AIS scores were stratified as follows: 0–3 points, 4–5 points, and ≥ 6 points. The distribution proportion of each absolute presenteeism scores was tabulated and the association between absolute presenteeism (≤ 40, > 40) and AIS (0–3 points, 4–5 points, ≥ 6 points) scores was examined using the chi-squared test. This method was also used to examine the relationship between relative presenteeism and AIS scores. The relationship between absolute presenteeism scores (cut-off value, 40 points) and shift work (day-only workers and shift workers) was examined. Percentage distribution of each absolute presenteeism score was tabulated and the association between the absolute presenteeism scores (≤ 40, > 40) and work schedule (day work only, shift work) was examined using the chi-squared test. This method was also used to examine the association between relative presenteeism scores and shift work. The cut-off value for presenteeism in a previous study correlated with the absolute/relative presenteeism score used in this present study (40/0.8). Similarly, the cut-off value for presenteeism correlated with future absence from work due to mental illness [28]. In addition, a large cohort epidemiological study of 45,000 participants reported a significant relationship between sleep disorders and future absence from work due to illness [29]. Considering the results of these two studies, we determined that the cut-off value determined in the previous study was sufficiently appropriate to examine the relationship between various states of sleep and presenteeism, which were the subjects of our study.

Next, we calculated the correlation coefficient using Spearman's rank correlation coefficient for the three categories of sleep duration, AIS score, and work schedule. A logistic regression analysis was conducted in which absolute presenteeism scores of ≤ 40 were defined as a high level of absolute presenteeism. A high level of absenteeism was regarded as an objective variable, and sleep duration, AIS score, and shift work were regarded as dependent variables. Also, the model included the following additional dependent variables: company, age, sex, type of work, position, type of employment, smoking, drinking, exercise, overtime, holidays, and the JPSS score. Forced entry was used as a variable selection method. Subjects with missing data were excluded from the analysis. Similarly, relative presenteeism scores of ≤ 0.8 were defined as a high level of relative presenteeism. A logistic regression analysis was conducted with a high level of relative presenteeism as an objective variable and sleep duration, AIS score, and shift work as dependent variables. Also, the model included the following additional dependent variables: company, age, sex, type of work, position, type of employment, smoking, drinking, exercise, overtime, holidays, and the JPSS score.

For all statistical analyses, the significance level was set at *P* < 0.05. The analyses were performed using IBM SPSS Statistics version 22.0 for Windows (IBM Corp., Armonk, NY, USA).

### Ethical considerations

The following ethical considerations were given in conducting this study. (1) Before the survey was conducted, the contents of the survey were explained in detail to company representatives at target offices, and consent to participate in the survey was obtained. (2) The content, purpose, and freedom to participate in the survey were fully explained to individual workers, and written informed consent was obtained from each participant. (3) The questionnaires were filled out by the individual workers and were subsequently submitted by the workers in a sealed envelope to ensure complete privacy. (4) This study was conducted with the prior approval from the ethics committees of universities with which the authors of this paper are affiliated (Nihon University School of Medicine Ethics Committee and Oita University Faculty of Medicine Ethics Committee). (5) This study was conducted in accordance with the “Ethical Guidelines for Medical and Health Research Involving Human Subjects” [30] established by the Ministry of Education, Culture, Sports, Science, and Technology and the Ministry of Health, Labour and Welfare.

## Results

### Participants’ number, basic characteristics, and score distribution of absolute/relative presenteeism

Completed questionnaires were collected from 1992 workers (response rate, 83.9%) and the data were included in the analysis. The characteristics and absolute/relative presenteeism score distributions of the participants whose data were analyzed are shown in Table 1. When incomplete data were excluded, men accounted for 74.8% and women accounted for 25.2% of the participants. The participants’ ages ranged from 18 to 79 years (average age ± standard deviation, 41.7 ± 11.8). In addition, as a result of, respectively, calculating the physical component summary (PCS) and mental component summary (MCS) scores using the SF-8®, the PCS average score was 47.1, and the MCS average score was 45.4 in the target population of this study. In 2007, the national standard values for PCS and MCS in Japan were 48.60 and 49.44, respectively [27]. However, it was determined that there was no significant difference between the average score and the national standard value concerning the medical condition in the population analyzed for this study. In this study, 40.7% of the respondents had an AIS score of 6 points or more. Similarly, a previous epidemiological study of 1666 local government employees living in Koka city, Japan, reported that 635 (38.1%) had an AIS score of 6 points or more [31], which was nearly the same prevalence as this study.

**Table 1:** Characteristics of workers in the study population and their score distributions of absolute/relative presenteeism

	*N*	%
Company		
Pharmaceutical company A	539	27.1
Automotive components manufacturer	765	38.4
Wholesaler of drugs	166	8.3
Pharmaceutical company B	94	4.7
Health service facility	138	6.9
IT company	290	14.6
Sex		
Male	1471	73.8
Female	496	24.9
Unknown	25	1.3
Age class		
≤ 29 y	385	19.3
30–39 y	462	23.2
40–49 y	560	28.1
50–59 y	454	22.8
≥ 60 y	105	5.3
Unknown	26	1.3
Type of work		
Desk work	379	19.0
Clerical work	1551	78.0
Unknown	62	3.1
Working position		
Non-managerial position	1673	84.0
Managerial position	251	12.6
Unknown	58	3.4
Type of employment		
Regular employment	1735	87.1
Irregular employment	218	10.9
Unknown	39	2.0
Smoking habit		
No or quit smoking	799	40.1
Sometimes	902	45.3
Everyday	115	5.8
Unknown	176	8.8
Drinking habit		
No or quit drinking	726	36.4
1 day/month–2 days/week	504	25.3
≥ 3 days/week	649	32.6
Unknown	113	5.7
Exercise habits		
I have been unable to exercise	1489	74.7
I have been exercising, but it has not become a habit	199	10.0
Exercising has become a habit	181	9.1
Unknown	123	6.2
Overtime hours in 1 month		
< 45 h	1643	82.5
≥ 45–< 80 h	178	8.9
≥ 80 h	17	0.9
Unknown	154	7.7
Holidays in 1 month		
< 4 d	247	12.4
4–7 d	451	22.6
8–11 d	991	49.7
≥ 12 d	168	8.4
Unknown	135	6.8
PSS score		
< 10 points	102	5.1
≥ 10–< 20 points	459	23.0
≥ 20–< 30 points	1102	55.3
≥ 30 points	148	7.4
Unknown	181	9.1
Sleep duration		
< 5 h/d	256	12.9
≥ 5–< 6 h/d	543	27.3
≥ 6–< 7 h/d	902	45.3
≥ 7–< 8 h/d	115	5.8
≥ 8–< 9 h/d	49	2.5
≥ 9 h/d	7	0.4
Unknown	120	6.0
AIS score		
0–3 points	686	34.4
4–5 points	330	16.6
≥ 6 points	811	40.7
Unknown	165	8.3
Working shift system		
Day work only	1180	59.2
Shift work	776	39.0
Unknown	36	1.8
Absolute presenteeism score (a lower score indicates poorer job performance by the individual worker and a higher level of absolute presenteeism)		
≤ 20	109	5.5
> 20– ≤ 40	291	14.6
> 40– ≤ 60	949	47.6
> 60– ≤ 80	467	23.4
> 80– ≤ 100	55	2.8
Unknown	121	6.1
Relative presenteeism score (a lower score indicates poorer job performance relative to other workers and a higher level of relative presenteeism)		
≤ 0.4	61	3.1
> 0.4– ≤ 0.8	283	14.2
> 0.8– ≤ 1.2	1184	59.4
> 1.2– ≤ 1.6	200	10.0
> 1.6– ≤ 2.0	95	4.8
Unknown	169	8.5

*IT* information technology; *PSS* Perceived Stress Scale; *AIS* Athens Insomnia Scale

Among the participants, 20.1% had an absolute presenteeism score of ≤ 40 (high level of absolute presenteeism; 95% confidence interval (CI) 18.3–21.9%) and 17.3% had a relative presenteeism score of ≤ 0.8 (high level of relative presenteeism; 95% CI 15.6–19.0%).

### Association of sleep duration, sleep quality, shift work, and absolute/relative presenteeism score

Table 2 shows the percentage distribution of absolute/relative presenteeism scores by sleep duration. Sleep duration was significantly associated with absolute presenteeism score (*P* = 0.007) but not with relative presenteeism score (*P* = 0.344).

**Table 2:** Associations of absolute/relative presenteeism and sleep duration (bivariate analysis)

	Absolute presenteeism score		*P*^a^	Relative presenteeism score		*P*^b^
	≤ 40 (poor performance)	> 40 (good performance)		≤ 0.8 (poor performance)	> 0.8 (good performance)	
Sleep duration			0.007			0.344
< 5 h/d						
Percentage	30.3	69.7		22.7	77.3	
Adjusted residual	3.8	− 3.8		1.6	− 1.6	
≥ 5–< 6 h/d						
Percentage	19.9	80.1		17.1	435	
Adjusted residual	− 0.8	0.8		− 1.3	1.3	
≥ 6–< 7 h/d						
Percentage	19.7	80.3		19.7	80.3	
Adjusted residual	− 1.4	1.4		0.8	− 0.8	
≥ 7–< 8 h/d						
Percentage	19.1	80.9		15.0	85.0	
Adjusted residual	− 0.5	0.5		− 1.1	1.1	
≥ 8–< 9 h/d						
Percentage	14.9	85.1		14.9	85.1	
Adjusted residual	− 1.1	1.1		− 0.7	0.7	
≥ 9 h/d						
Percentage	28.6	71.4		28.6	71.4	
Adjusted residual	0.5	− 0.5		0.7	− 0.7	

Missing data were excluded from the statistical analyses

^a^*P* was calculated by chi-squared test. 2 absolute presenteeism score (< 40, > 40) × 6 sleep duration (< 5 h/d, > 5–< 6 h/d, > 6–< 7 h/d, > 7–< 8 h/d, > 8–< 9 h/d, > 9 h/d)

^b^*P* was calculated by chi-squared test. 2 relative presenteeism score (< 0.8, > 0.8) × 6 sleep duration (< 5 h/d, > 5–< 6 h/d, > 6–< 7 h/d, > 7–< 8 h/d, > 8–< 9 h/d, > 9 h/d)

Table 3 shows the percentage distribution of absolute/relative presenteeism score per AIS score interval. AIS score was significantly associated with absolute presenteeism score (*P* < 0.001) and relative presenteeism score (*P* < 0.001).

**Table 3:** Associations of absolute/relative presenteeism and AIS score (bivariate analysis)

	Absolute presenteeism Score		*P*^a^	Relative presenteeism score		*P*^b^
	≤ 40 (poor performance)	> 40 (good performance)		≤ 0.8 (poor performance)	> 0.8 (good performance)	
AIS score			< 0.001			< 0.001
0–3 points						
Percentage	14.1	85.9		14.1	85.9	
Adjusted residual	− 5.5	5.5		− 4.0	4.0	
4–5 points						
Percentage	21.2	78.8		22.0	78.0	
Adjusted residual	0.2	− 0.2		1.6	− 1.6	
≥ 6 points						
Percentage	26.5	73.5		21.6	78.4	
Adjusted residual	5.3	− 5.3		2.6	− 2.6	

Missing data were excluded from the statistical analyses

*AIS* Athens Insomnia Scale

^a^*P* was calculated by chi-squared test. 2 absolute presenteeism score (< 40, > 40) × 3 AIS score (0–3, 4–5, > 6)

^b^*P* was calculated by chi-squared test. 2 relative presenteeism score (< 0.8, > 0.8) × 3 AIS score (0–3, 4–5, > 6)

Table 4 shows the percentage distribution of absolute/relative presenteeism scores by work schedule. Shift work was not significantly associated with absolute presenteeism score (*P* = 0.111) but was significantly associated with relative presenteeism score (*P* = 0.003).

**Table 4:** Associations of absolute/relative presenteeism and shift work (bivariate analysis)

	Absolute presenteeism score		*P*^a^	Relative presenteeism score		*P*^b^
	≤ 40 (poor performance)	> 40 (good performance)		≤ 0.8 (poor performance)	> 0.8 (good performance)	
Working shift system			0.111			0.003
Day work only						
Percentage	22.7	77.3		21.2	78.8	
Adjusted residual	1.6	− 1.6		3.0	− 3.0	
Shift work						
Percentage	19.6	80.4		15.5	84.5	
Adjusted residual	− 1.6	1.6		-3.0	3.0	

Missing data were excluded from the statistical analyses

^a^*P* was calculated by chi-squared test. 2 absolute presenteeism score (< 40, > 40) × 2 working shift system (day work only, shift work)

^b^*P* was calculated by chi-squared test. 2 relative presenteeism score (< 0.8, > 0.8) × 2 working shift system (day work only, shift work)

The correlation coefficients were calculated for each of the three categories: sleep duration, AIS score, and work schedule. The correlation coefficient between sleep duration and AIS score was − 0.247 (*P* < 0.001), the correlation coefficient between AIS score and work schedule was 0.110 (*P* < 0.001), and the correlation coefficient between work schedule and sleep duration was 0.023 (*P* = 0.317).

Table 5 shows the results of the logistic regression analysis in which a high level of absolute/relative presenteeism was regarded as an objective variable, and sleep duration, sleep quality (AIS score), and shift work were regarded as dependent variables. Absolute presenteeism score was significantly associated with poor sleep quality (high AIS score; *P* < 0.001), but not with sleep duration (*P* = 0.326) or shift work (*P* = 0.177). A high level of relative presenteeism was significantly associated with poor sleep quality (high AIS score; *P* = 0.001), but not with sleep duration (*P* = 0.461) or shift work (*P* = 0.245).

**Table 5:** Associations between absolute/relative presenteeism and various factors (including sleep duration, AIS, and shift work) (multivariate analysis)

	High level of absolute presenteeism^a^ (poor absolute job performance)					High level of relative presenteeism^b^ (poor relative job performance)				
	COR	95% CI	AOR	95% CI	*P*^c^	COR	95% CI	AOR	95% CI	*P*^c^
Company										
Pharmaceutical company A	1.00		1.00			1.00		1.00		
Automotive components manufacturer	1.17	0.88–1.57	1.32	0.90–1.94		0.73	0.54–0.98	0.90	0.60–1.36	
Wholesaler of drugs	0.83	0.51–1.34	0.55	0.29–1.02		0.78	0.48–1.25	0.49	0.26–0.90	
Pharmaceutical company B	0.78	0.42–1.44	0.75	0.36–1.56		0.82	0.46–1.47	0.94	0.47–1.90	
Health service facility	1.16	0.72–1.88	1.28	0.72–2.25		0.92	0.56–1.51	1.02	0.57–1.83	
IT company	2.07	1.48–2.90	1.34	0.82–2.18		1.47	1.03–2.09	1.43	0.87–2.35	
Sex					0.610					0.367
Male	1.00		1.00			1.00		1.00		
Female	1.26	0.98–1.62	0.92	0.66–1.28		1.44	1.11–1.87	1.17	0.83–1.64	
Age class					< 0.001					< 0.001
≤ 29 y	1.00		1.00			1.00		1.00		
30–39 y	0.59	0.43–0.81	0.51	0.35–0.74		0.62	0.44–0.86	0.51	0.34–0.75	
40–49 y	0.55	0.40–0.74	0.51	0.35–0.75		0.54	0.39–0.74	0.45	0.30–0.67	
50–59 y	0.40	0.29–0.57	0.42	0.27–0.64		0.48	0.34–0.68	0.46	0.30–0.72	
≥ 60 y	0.18	0.09–0.39	0.14	0.05–0.38		0.23	0.11–0.48	0.13	0.05–0.35	
Type of work					0.196					0.257
Desk work	1.00		1.00			1.00		1.00		
Clerical work	0.87	0.66–1.15	0.78	0.54–1.14		0.84	0.63–1.13	0.80	0.54–1.18	
Working position					0.020					0.101
Non-managerial position	1.00		1.00			1.00		1.00		
Managerial position	0.54	0.37–0.79	0.56	0.34–0.91		0.60	0.40–0.89	0.66	0.40–1.09	
Type of employment					0.387					0.155
Regular employment	1.00		1.00			1.00		1.00		
Irregular employment	0.69	0.47–1.02	0.80	0.48–1.32		1.32	0.93–1.88	1.41	0.88–2.26	
Smoking habit					0.947					0.507
No or quit smoking	1.00		1.00			1.00		1.00		
Sometimes	0.90	0.39–2.08	1.12	0.43–2.87		0.37	0.11–1.23	0.50	0.15–1.73	
Everyday	0.76	0.58–0.98	0.97	0.72–1.31		0.72	0.55–0.95	0.92	0.67–1.27	
Drinking habit					0.220					0.141
No or quit drinking	1.00		1.00			1.00		1.00		
1 day/month–2 days/week	0.90	0.69–1.18	0.85	0.62–1.16		0.93	0.70–1.23	0.87	0.63–1.20	
≥ 3 days/week	0.56	0.43–0.74	0.76	0.55–1.05		0.54	0.41–0.73	0.71	0.50–1.00	
Exercise habits					0.525					0.139
I have been unable to exercise	1.00		1.00			1.00		1.00		
I have been exercising, but it has not become a habit	0.80	0.54–1.17	0.90	0.59–1.36		0.89	0.60–1.31	0.87	0.56–1.35	
Exercising has become a habit	0.78	0.52–1.17	0.77	0.48–1.24		0.66	0.42–1.03	0.60	0.36–1.00	
Overtime hours in one month					0.321					0.728
< 45 h	1.00		1.00			1.00		1.00		
≥ 45–< 80 h	1.65	0.36–7.62	1.62	0.32–8.27		0.85	0.23–3.18	0.71	0.16–3.17	
≥ 80 h	1.87	0.42–8.25	2.15	0.44–10.48		0.94	0.26–3.34	0.85	0.20–3.60	
Holidays in one month					0.081					0.016
< 4 d	0.73	0.46–1.17	0.67	0.38–1.15		0.41	0.25–0.69	0.51	0.28–0.92	
4–7 d	0.63	0.41–0.96	0.52	0.31–0.83		0.43	0.28–0.67	0.49	0.28–0.84	
8–11 d	0.78	0.53–1.13	0.75	0.48–1.17		0.74	0.51–1.08	0.80	0.51–1.25	
≥ 12 d	1.00		1.00			1.00		1.00		
PSS score					0.110					0.111
< 10 points	1.00		1.00			1.00		1.00		
≥ 10–< 20 points	0.97	0.54–1.75	0.82	0.42–1.59		1.22	0.63–2.35	0.96	0.47–1.99	
≥ 20–< 30 points	1.53	0.88–2.66	1.22	0.65–2.30		1.87	1.00–3.48	1.37	0.68–2.74	
≥ 30 points	1.88	0.98–3.59	1.21	0.57–2.57		1.76	0.84–3.65	0.94	0.41–2.17	
Sleep duration					0.326					0.461
< 5 h/d	2.48	1.06–5.79	2.28	0.82–6.39		1.68	0.71–3.96	1.14	0.42–3.08	
≥ 5– < 6 h/d	1.42	0.62–3.26	1.50	0.55–4.10		1.18	0.51–2.72	1.03	0.40–2.68	
≥ 6–< 7 h/d	1.40	0.62–3.18	1.58	0.59–4.25		1.40	0.62–3.18	1.36	0.53–3.45	
≥ 7–< 8 h/d	1.35	0.54–3.42	1.62	0.54–4.89		1.01	0.39–2.63	0.83	0.28–2.47	
≥ 8–< 9 h/d	1.00		1.00			1.00		1.00		
≥ 9 h/d	2.29	0.37–14.19	1.66	0.24–11.77		2.29	0.37–14.19	1.19	0.17–8.22	
AIS score					< 0.001					0.001
0–3 points	1.00		1.00			1.00		1.00		
4–5 points	1.65	1.17–2.32	1.67	1.14–2.45		1.71	1.22–2.41	1.82	1.24–2.67	
≥ 6 points	2.21	1.69–2.88	2.09	1.52–2.87		1.68	1.27–2.21	1.78	1.28–2.47	
Working shift system					0.177					0.245
Day work only	1.00		1.00			1.00		1.00		
Shift work	0.83	0.66–1.04	0.78	0.54–1.12		0.68	0.53–0.88	0.80	0.55–1.17	

^a^Objective variable: High level of absolute presenteeism (absolute presenteeism score: ≤ 40)

^a^Objective variable: High level of relative presenteeism (relative presenteeism score: ≤ 0.8)

^c^*P* was calculated by multiple logistic regressions (forced entry method)

Missing data were excluded from the statistical analyses

*COR* crude odds ratio; *AOR* adjusted odds ratio; *CI* confidence interval; *PSS* Perceived Stress Scale; *AIS* Athens Insomnia Scale

## Discussion

This epidemiological study investigated whether workers’ presenteeism is associated with sleep duration, sleep quality, and shift work. Although several previous studies have established an association between presenteeism and sleep, this is the first study to examine the association between workers’ presenteeism and three components of sleep (quantity, quality, and rhythm) simultaneously. Furthermore, the relationship between sleep quality and presenteeism was examined quantitatively using the AIS.

Here, we established a statistically significant association between short sleep duration and the aggravation of presenteeism. Numerous studies have reported that sleep duration is associated with productivity and presenteeism [10–12, 32]. In an epidemiological study of 710 Australian workers, no significant linear relationship was established between sleep duration and presenteeism after adjusting for all lifestyle behaviors and sociodemographic factors (standardized regression coefficient (*B*) = 0.081; *P* < 0.05). In the study, sleep durations of < 7 or ≥ 8 h were reported to adversely affect presenteeism and 7 h of sleep was reported to be the most favorable sleep duration, corresponding to the “*U*-shaped curve” [32]. A previous epidemiological study established that short sleep duration was associated with the development of various lifestyle-relate diseases [33]. We hypothesized that sleep duration and presenteeism have a *U*-shaped curve relationship and thus conducted this study. The results of logistic regression analysis showed that for absolute presenteeism, the AOR increased when sleep duration was shorter or longer than the standard sleep duration of > 8 to ≤ 9 h/d, which was in the form of a U-shaped curve. However, the relationship between the two was not statistically significant. The reason for the lack of statistical significance in this study may be the problem of sample size. In particular, according to the international comparative statistics of sleep duration (OECD Gender Data Portal), it is known that Japanese people sleep much lesser compared with people in other countries [34], and the number of those who fall into the category of long sleepers, in particular, is expected to be small. In this study, only 0.9% of the participants had a sleep duration of 9 h or longer, which is extremely low, and the detection power may have been low. Therefore, it will be necessary to consider designing a study with a larger sample size in the future.

This study established a significant association between sleep quality (as evaluated using the AIS) and presenteeism (poor sleep quality and aggravation of presenteeism). A previous cross-sectional epidemiological study showed a significant association between subjective sleep quality and presenteeism after adjusting for lifestyle behaviors and sociodemographic factors, including sleep duration [32]. This study used the Pittsburgh Sleep Quality Index (PSQI) [35] to assess sleep quality. However, only one of the 17 questions comprising the PSQI was used to assess sleep quality (the remaining 16 questions were not used to assess sleep quality) [32]. Therefore, it may not have been an adequate assessment of comprehensive sleep quality. In this study, sleep quality was assessed using all the 8 questions of the AIS—a comprehensive sleep quality assessment scale with verified adequate reliability and validity. Our findings, therefore, provide more reliable evidence for the relationship between sleep quality and presenteeism suggested by previous studies.

The univariate analyses conducted in the present study showed that shift work is statistically significantly associated with the relative presenteeism score but not with the absolute presenteeism score. Furthermore, multivariate analyses showed that neither absolute nor relative presenteeism were significantly associated with shift work. A previous study had shown that shift work disrupts a normal circadian rhythm, causing the so-called “shift-lag syndrome” characterized by increased fatigue, sleepiness, insomnia, disorientation, digestive troubles, irritability, poorer mental agility, and reduced performance efficiency [36]. We hypothesized that the shift-lag syndrome is an independent factor that adversely affects presenteeism, as it is thought to lead to the deterioration of health and work performance. However, in a multivariate analysis adjusted for other factors, no significant association was established between shift work and presenteeism. A previous epidemiological study of 725 Finnish workers reported that there was no significant association between shift work and presenteeism [37]. By contrast, a multivariate analysis conducted in an epidemiological study of 6220 South Korean workers showed a significant association between shift work and presenteeism [38]. This difference may be attributed to differences in study population or the adjustment factors in the multivariate analysis. Moreover, both studies had a major limitation in that sleep duration and sleep quality were not considered as adjustment factors in examining the relationship between shift work and presenteeism. As shift work has been shown to be associated with sleep duration and sleep quality [39], these factors may need to be adjusted for when investigating associations between shift work and presenteeism. To date, a relationship between shift work and presenteeism has not been established, and more research on this is required.

The present study has several limitations. Sleep duration and sleep quality were evaluated through self-administered questionnaires, which provided a subjective rather than objective method of measurement such as polygraphs or actigraphs. Hence, the physiological mechanism of the relationship between sleep and presenteeism has not been clarified. In addition, in this study, while sleep rhythm was evaluated by "day work only" or "shift work," the difference between "day work only" and "shift work" does not mean the difference between healthy and disordered. Therefore, there is a possibility that sleep rhythm disorder will not be assessed appropriately. If the definition is not established correctly, there may be a serious change in the results. In future research, it will be desirable to utilize scales that have been proven to have sufficient reliability and validity with appropriate cut-off points for sleep rhythm. Furthermore, as this is a cross-sectional study, we cannot discuss the causality in the relationship between sleep and presenteeism [40]. A longitudinal study is needed to resolve this limitation and to establish a causal relationship.

Here, we showed that sleep duration and sleep quality are associated with presenteeism. This indicates that improving workers’ sleep duration and sleep quality may improve productivity and reduce presenteeism. In the future, it may be necessary to focus on promoting sleep improvement among workers when considering social policies aimed at increasing workers’ productivity. In addition to addressing this issue at the individual level, it is also necessary to introduce mechanisms and initiatives aimed at promoting sleep improvement in society as a whole. Moreover, it would be desirable to conduct further academic research, such as longitudinal observational studies aimed at obtaining further evidence—including proof of a causal relationship between sleep and presenteeism—and intervention studies to examine whether sleep improvement would actually reduce presenteeism.
